# Pituitary macroadenoma resulting from primary hypothyroidism; a 16-year-old girl

**DOI:** 10.22088/cjim.10.3.347

**Published:** 2019

**Authors:** Zahra Davoudi, Arezoo Chouhdari, Omidvar Rezaei, Guive Guive

**Affiliations:** 1 Skull Base Research Center, Loghman Hakim Medical Center, Shahid Beheshti University of Medical Sciences, Tehran, Iran; 2 Department of Neurosurgery, Loghman Hakim Hospital, Shahid Beheshti University of Medical Sciences, Tehran, Iran

**Keywords:** Primary hypothyroidism, Pituitary macro adenoma, Galactorrhea

## Abstract

**Background:**

Primary hypothyroidism is a common medical condition. It can lead to pituitary adenoma which is usually asymptomatic, but it can also lead to symptomatic macroadenomas which are hard to diagnose due to different clinical presentations.

**Case presentation:**

A 16-year-old girl presented for endocrinology consultation prior to neurosurgical operation. She had galactorrhea which was accompanied by vertigo & low grade blurred vision without a headache and was diagnosed with pituitary macroadenoma and was planned for a surgery. She had TSH level of more than 100 mU/L, free thyroxine of 1.9 pmol/L. Her thyroid peroxidase (TPO) antibody level was 13.3 IU/mL, insulin growth factor-1 392 µ/l and serum prolactin level 42 ng/ml. During physical exam and with the laboratory findings, we suspected for a primary hypothyroidism as the leading cause of pituitary macroadenoma. As the result, we cancel the surgery and start levothyroxine therapy 100µg daily for her. In the follow-up it revealed that our diagnosis was correct and she went into remission with pituitary gland shrinking and decreasing TSH and prolactin levels.

**Conclusion:**

It is important to understand the different presentation of primary hypothyroidism to decrease the unnecessary risk of maltreatment in patients.

Primary hypothyroidism or thyroid hormone deficiency due to the abnormality in the thyroid gland is the most common endocrine disease. The prevalence of hypothyroidism in the general population ranges from 3.8%–4.6 (1). Symptoms may be vague, misleading and thus, delay the diagnosis, which can be made in unusual circumstances. Thyrotrophic cell hyperplasia has been described in longstanding hypothyroidism and may mimic the appearance of a pituitary macroadenoma (2, 3). Pituitary hyperplasia is an enlargement of the pituitary gland due to a reversible increase in the number and/or hyperplasia in one or more hormone-producing cell types. It can occur as a normal response to physiological stimulation during infancy, pregnancy, and lactation, or as a pathological condition (2). Pituitary enlargement in primary hypothyroidism is mainly asymptomatic (4). We are presenting a case of primary hypothyroidism which led to symptomatic pituitary enlargement and the patient was at risk of the unnecessary neurosurgical operation.

## Case presentation

A 16-year-old female presented to Endocrinology clinic for pre-surgical consultation. She was a candidate for neurosurgical operation due to hypophyseal macroadenoma.

**Figure 1 (A: left, B: Right):**
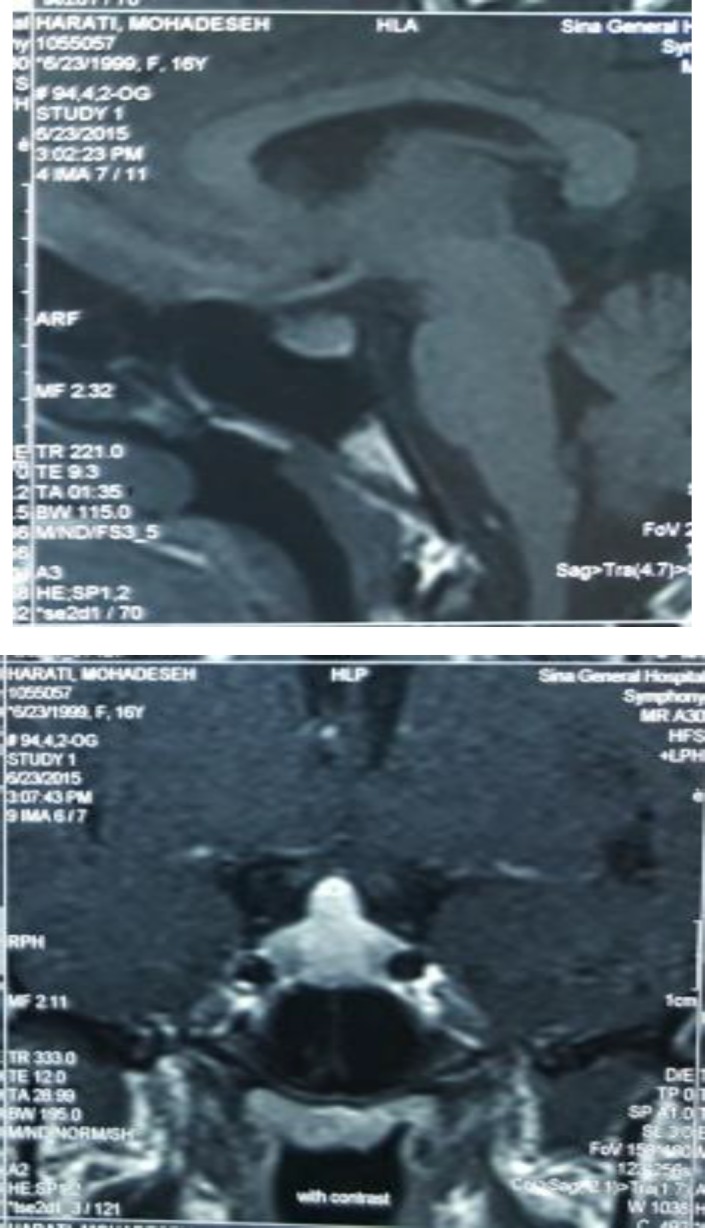
First MRI before treatment showed a 17×12×11 mm pituitary mass

Her problems began 7 months ago with a chief complaint of galactorrhea which was accompanied by vertigo and low grade blurred vision without any headache. She had regular menstruations and had her first menstruation when she was 12 years old. She had no significant family history, she did not use any drugs with normal habitual history. She was married but she did not have children. In her primary visit, laboratory tests were asked for her and the following measures were reported: CBC ,ESR showed normal measures , thyroid stimulation hormone (TSH) level of more than 100 mU/L (reference range 0.350–4.94), free thyroxine (FT4) of 2.4 pmol/L (9.0–19.0 ), serum cortisol level was 14 µ g/dl (3-20 µg/dl), insulin growth factor-1 (IGF-1) 235 µ/l (84–100 µ/l) and serum prolactin level 31 ng/ml (4-23 ng/mL). Based on the above laboratory investigation, she was referred for a neurosurgical consultation due to her vertigo. Neurosurgeon asked for a brain MRI which showed the following measures: 

Pituitary magnetic resonance imaging (MRI) revealed a 17×12×11 mm homogeneous pituitary mass with upward convexity, discreetly hyper-intense on a T1 weighted image and hyper-intense on a T2 weighted image too (figure 1).

 A neurosurgical operation was decided for the patient and she referred for endocrinology consultation. In endocrinology clinic (Loghman Hakim Hospital), physical examination revealed a well-developed female. Her weight was 54 kg, height 157cm and her body mass index was 22. Her vital signs were stable. She had normal secondary sexual characteristics; in thyroid examination, we found a diffuse goiter with 30-gram weight and firm consistency and induced galactorrhea was seen, too.

New necessary laboratory tests considering previous laboratory tests were requested for her in our endocrinology clinic which showed the following measures: 

Thyroid stimulation hormone (TSH) level of more than 100 mU/L (reference range 0.350–4.94), free thyroxine (FT4) of 1.9 pmol/L (9.0–19.0). Her thyroid peroxidase (TPO) antibody level was 13.3 IU/mL (less than 35 IU/mL, serum cortisol level was 15.8 µg/dl (3-20 µg/dl), insulin growth factor-1 (IGF-1) 392 µ/l (84–100 µ/l) and serum prolactin level 42 ng/ml (4-23 ng/ml. She had normal perimetry (visual field test) although with blurred vision.

## Discussion

The main complaint of our patient was a galactorrhea which was due to high prolactin level. Fatigue and vertigo due to pituitary macro-adenoma led to performing cerebral imaging. The clinical features of hypothyroidism were unmarked in this case which leads to misdiagnosis in the first step. Primary hypothyroidism can result in reactive enlargement of the pituitary gland which is indistinguishable from primary pituitary lesions on magnetic resonance imaging (MRI) and these results from the loss of thyroxine feedback inhibition and subsequent over-production of thyroid-stimulating hormone (5). It is important to differentiate between reactive enlargement of the pituitary gland due to longstanding hypothyroidism from non-function pituitary macroadenoma that is usually presented with hypopituitarism and hyperprolactinemia due to stalk effect. There is a low level of FT4 and low TSH. In non-function pituitary macroadenoma, not any high TSH is compatible with our case (6) and so is lymphocytic hypophysitis. An unusual postpartum lymphocytic inflammatory pituitary lesion can be associated with a mass lesion. Over half patients with lymphatic hypophysitis present with headache, visual field impairment and hyperprolactinemia. 56% of patients have secondary hypoadrenalism, followed in frequency by hypothyroidism, hypogonadism, and GH deficiency. The pituitary stalk may thicken especially when diabetes insipidus is present. Erythrocyte sedimentation rate (ESR) is often elevated Although in this case is not fulfilled criteria of lymphocytic hypophysitis, but maybe concomitantly both diseases are not known (7) and finally with regard to TSH –producing adenoma (8) that our patient had no symptoms or signs of hyperthyroidism such as palpitation, tremor, and there was no elevation in FT4 and FT3 in lab tests that was compatible with TSH -producing adenoma.

**Figure 2 (A: left, B: right):**
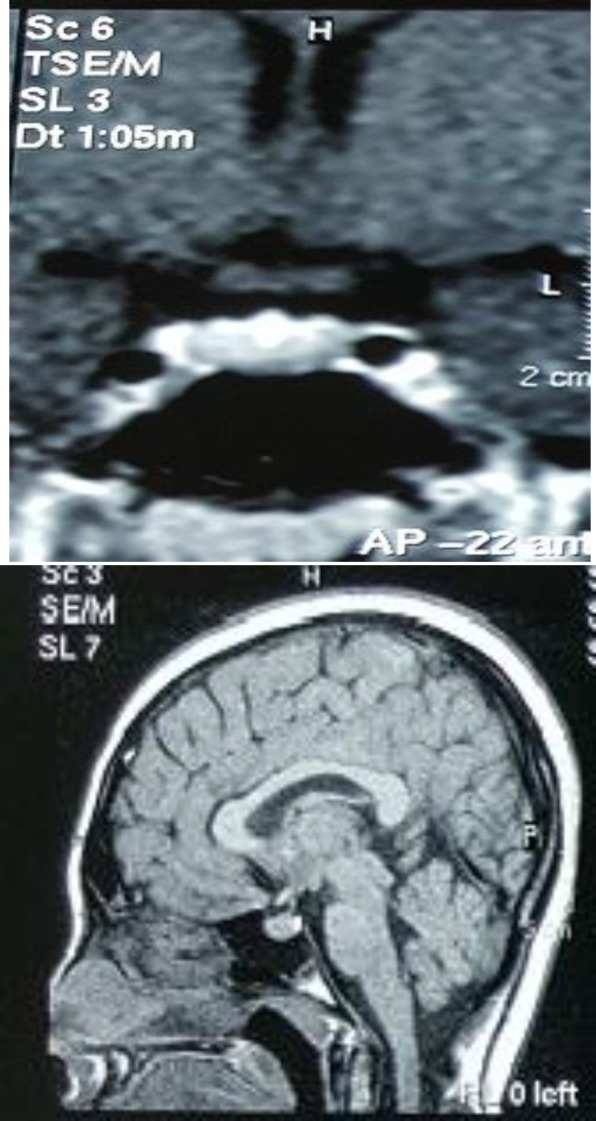
MRI (4 months after treatment) showed a small (2 mm) lesion at left pituitary lobe

Based on the clinical, laboratory and imaging findings, we considered a diagnosis of primary hypothyroidism complicated by compensatory pituitary hyperplasia. With this diagnosis, we decided to cancel surgery and started the patient on levothyroxine substitution therapy gradually increasing to 100 µg daily, after 2 months follow-up, his TSH level of 0.6 mU/L (reference range 0.350–4.94), FT4 6.7 pmol/L (9.0–19.0) and serum prolactin level of 16 ng/ml (4-23 ng/mL) were in normal reference ranges; galactorrhea and blurred vision disappeared. Follow-up MRI study of 4 months later documented "a small (2mm) pituitary over shape lesion at left pituitary lobe " and 1 year later, no pituitary mass was reported (figure 2).

Considering the significant decrease in tumor size, the diagnosis was made as thyrotrophic hyperplasia in the pattern of primary hypothyroidism and the patient did not need neurosurgical treatment and answered well to levothyroxine, according to our case, Sarliset al. reported a dramatic shrinkage of a pituitary mass in a case of primary hypothyroidism after only one week of acute thyroid hormone therapy which make us more comfortable with our decision (9) also, Amal Moumenet al. reported a similar case of pituitary macroadenoma due to primary hypothyroidism that responded perfectly to levothyroxine therapy with great shrinkage of pituitary mass after 4 months of treatment which is exactly the same as our case (10).

In conclusion, based on the results of this case, although there was enough knowledge of the patient's hormonal profile, the pituitary mass was first reported as a macroadenoma. After documenting the primary hypothyroidism, we considered the diagnosis of reactive pituitary hyperplasia, which was definitively confirmed by the regression of the pituitary mass on the control MRI after thyroxine therapy allowing us to avoid an unnecessary surgery.
